# Shotgun proteomics analysis of SARS-CoV-2-infected cells and how it can optimize whole viral particle antigen production for vaccines

**DOI:** 10.1080/22221751.2020.1791737

**Published:** 2020-07-27

**Authors:** Lucia Grenga, Fabrice Gallais, Olivier Pible, Jean-Charles Gaillard, Duarte Gouveia, Hélène Batina, Niza Bazaline, Sylvie Ruat, Karen Culotta, Guylaine Miotello, Stéphanie Debroas, Marie-Anne Roncato, Gérard Steinmetz, Charlotte Foissard, Anne Desplan, Béatrice Alpha-Bazin, Christine Almunia, Fabienne Gas, Laurent Bellanger, Jean Armengaud

**Affiliations:** Département Médicaments et Technologies pour la Santé (DMTS), Université Paris Saclay, CEA, INRAE, SPI Bagnols-sur-Cèze, France

**Keywords:** COVID-19, SARS-CoV-2, proteomics, host response, infection kinetics, vaccine, mass spectrometry, viral protein detection

## Abstract

Severe acute respiratory syndrome-related coronavirus 2 (SARS-CoV-2) has resulted in a pandemic and is continuing to spread rapidly around the globe. No effective vaccine is currently available to prevent COVID-19, and intense efforts are being invested worldwide into vaccine development. In this context, all technology platforms must overcome several challenges resulting from the use of an incompletely characterized new virus. These include finding the right conditions for virus amplification for the development of vaccines based on inactivated or attenuated whole viral particles. Here, we describe a shotgun tandem mass spectrometry workflow, the data produced can be used to guide optimization of the conditions for viral amplification. In parallel, we analysed the changes occurring in the host cell proteome following SARS-CoV-2 infection to glean information on the biological processes modulated by the virus that could be further explored as potential drug targets to deal with the pandemic.

## Introduction

Closely related to the SARS-CoV virus, SARS-CoV-2 is a B lineage beta-coronavirus [1,2]. It is the causative agent of COVID-19, a severe acute respiratory syndrome that spread worldwide within a few weeks following its initial emergence in Wuhan (China) in December 2019 [3]. The four major structural genes encode the nucleocapsid protein (N), the spike protein (S), an envelope protein (E) and the membrane glycoprotein (M); an additional membrane glycoprotein (HE) is present in the HCoV-OC43 and HKU1 beta-coronavirus genus [3].

Based on the speed at which the outbreak of COVID-19 developed, SARS-CoV-2 spreads easily among human populations. The reproductive number (*R*_0_) for the uncontained virus is currently thought to be around 3, suggesting a potential for sustained human-to-human transmission that appears to occur through exchange of respiratory droplets, and possibly a faecal-oral route [4]. In this pandemic situation, one of the outstanding questions relates to how we can contain the spread of SARS-CoV-2 and its persistence in the human population. Social distancing policies, lockdown, and other containment measures have been implemented worldwide to slow the spread of the pandemic. Current roadmaps to lifting these restrictions hinge on the deployment of effective diagnostic and therapeutic strategies, and ultimately on the development of an effective vaccine.

Several platforms are being used to develop SARS-CoV-2 vaccines, approaches include attempts to produce spike subunit, DNA, RNA, whole-virion, and nanoparticle vaccines [5]. Most successful vaccines use inactivated or attenuated whole viral particles as vaccine antigen to induce neutralizing antibodies against viral structural proteins [6–8]. However, virus yields from the dedicated cell culture systems may be relatively low compared to the quantities expected to be required for massive vaccine production. In addition, virus production campaigns are time-consuming and highly demanding due to the danger of working with these pathogens. As a result, optimization of the production of whole viral particle antigens is of great interest in the search for effective vaccines. Alongside vaccine development, a better understanding of how the host responds to SARS-CoV-2 infection may help direct further therapeutic avenues.

Multiple proteomics strategies have improved our understanding of the structures of coronaviruses and the molecular mechanisms they exploit for infection. Tracheal tissues from chickens infected with infectious bronchitis coronavirus were analysed by 2D-DIGE and MALDI-TOF tandem mass spectrometry to establish the host response [9]. Vero cells infected with porcine epidemic diarrhoea virus (PEDV) were analysed by shotgun proteomics [10]. Several PEDV coronavirus strains were compared using a quantitative approach, i.e. iTRAQ-labelling [11]. The results revealed differences in elicitation of the inflammatory cascade. The dynamics of the host proteins affected by specific overexpressed coronavirus genes was also established [12]. Although no study has yet been published on the proteomics characterization of SARS-CoV-2 virus, several manuscripts of interest were recently submitted and should soon become available [13–15].

Here, we describe a workflow based on shotgun tandem mass spectrometry data that, in addition to gaining more basic information about SARS-CoV-2 infection, aims to guide the optimization of the conditions for whole viral particle antigen production and aid SARS-CoV-2 vaccine development.

## Materials and methods

### Cell culture and virus preparation

Vero E6 (ATCC, CLR-1586) cells were cultured at 37°C under 9% CO_2_ in Dulbecco's modified Eagle's medium (DMEM, Gibco™, ThermoFisher) supplemented with 5% foetal calf serum (FCS) and 0.5% penicillin–streptomycin. Cells were passaged following trypsinization every 2 days. The SARS-CoV-2 strain 2019-nCoV/Italy-INMI1 (Genbank MT066156) was provided by the Lazzaro Spallanzani National Institute of Infectious Diseases (Rome, Italy) via the EVAg network (European Virus Archive goes global). The stock SARS-CoV-2 used in the experiments described here had undergone two passages on Vero E6 cells and was stored at −80°C. Virus titre was determined by standard plaque assay (1 × 10^7^ pfu/ml).

### Infection

To determine the kinetics of infection, 1 × 10^6^ Vero cells seeded in 25 cm^2^ flasks were grown to confluence in 5 mL DMEM supplemented with 5% FCS and 0.5% penicillin–streptomycin overnight at 37°C under 9% CO_2_. Cells were infected at two multiplicities of infection (MOI; 0.01 and 0.001) and harvested at 1, 2, 3, 4 and 7 days post-infection (dpi). Supernatants from SARS-CoV-2-infected cells were recovered for plaque assay titration to confirm the production of infectious viral particles. Infected Vero cells were observed under a microscope to determine cytopathic effect at the same time-points.

### Quantification of viral RNA by RT-qPCR

SARS-CoV-2 RNA from cell culture supernatant samples, and supernatant plus cells was isolated using the NucleoSpin RNA Virus Mini kit (Macherey Nagel) according to the manufacturer’s instructions. The kit is designed to recover viral RNA from cell-free fluids. RNA was analysed by RT-qPCR using the SuperScript III Platinum One-Step RT-qPCR Kit (ThermoFisher) and a CFX96 Touch Real-Time PCR Detection System Thermal Cycler (BioRad). Primers targeting IP2 and IP4 (RdRp), used at 0.4 µM per reaction, were as recommended [16]: nCoV_IP2-12669Fw (ATGAGCTTAGTCCTGTTG) nCoV_IP2-12759Rv (CTCCCTTTGTTGTGTTGT) nCoV_IP2-12696bProbe(+) (AGATGTCTTGTGCTGCCGGTA [5’]Hex [3’]BHQ-1) and nCoV_IP4-14059Fw (GGTAACTGGTATGATTTCG) nCoV_IP4-14146Rv (CTGGTCAAGGTTAATATAGG) nCoV_IP4-14084Probe(+) (TCATACAAACCACGCCAGG [5’]Fam [3’]BHQ-1), respectively. Standard curves were created using *in vitro*-transcribed RNA derived from the BetaCoV_Wuhan_WIV04_2019 strain (EPI_ISL_402124). The transcript contains the amplification regions of the RdRp and E gene as positive strand. Microtubes were prepared with 1011 copies of target sequences diluted in yeast tRNAs (to facilitate recovery), and lyophilized. Mean and standard deviation were calculated for each group (*n* = 3).

### Sample preparation for mass spectrometry

At several time-points, SARS-CoV-2-infected Vero cells were washed twice with 5 ml of PBS to remove cell culture medium and FCS. Cells were recovered in 1.5 ml of PBS. The virus was inactivated and the cells lysed by autoclaving the samples at 125°C for 40 min. Proteins were precipitated by adding cold trichloroacetic acid to a final concentration of 10% (w/v). After 5 min at 4°C, the precipitated material was recovered by centrifugation at 16,000 *g* for 10 min. The proteins in the resulting pellets were dissolved in 100 μL LDS 1X (Lithium dodecyl sulfate) sample buffer (Invitrogen) supplemented with 5% beta-mercaptoethanol (vol/vol). Samples were sonicated with a Hielscher UP50H disruptor for 20 s operating at 60% amplitude, alternating 0.25-s impulsions with incubation for 5 min at 99°C. A 20-µl aliquot of a 1/8 dilution in LDS 1X (Invitrogen) of each sample was loaded on a NuPAGE 4–12% Bis-Tris gel and subjected to short (5-min) SDS-PAGE migration. Proteins were stained for 5 min with Coomassie SimplyBlue SafeStain (Thermo Fisher Scientific) prior to in-gel trypsin proteolysis, as described in Hartmann et al. [17].

### Liquid chromatography-mass spectrometry

Peptides were identified using an ultimate 3000 nano-LC system (Thermo Fisher Scientific) coupled to a Q-Exactive HF mass spectrometer (Thermo Fisher Scientific). Peptides were first desalted on a reverse-phase PepMap 100 C18 μ-precolumn (5 μm, 100 Å, 300 μm i.d. × 5 mm, Thermo Fisher Scientific) before separation on a nanoscale PepMap 100 C18 nano-LC column (3 μm, 100 Å, 75 μm i.d. × 50 cm, Thermo Fisher Scientific) by applying a 120 min gradient (100 min from 4% to 25% solvent B, and 20 min from 25% to 40% of solvent B) at a flow rate of 0.2 μL per min. Solvent A was 0.1% formic acid in water, solvent B was 80% acetonitrile, 0.1% formic acid in water. The mass spectrometer was operated in Top20 mode. Full MS were acquired from 350 to 1500 *m/z* and the 20 most abundant precursor ions were selected for fragmentation, applying a 10-s dynamic exclusion window. Ions with charge 2+ and 3+ were selected for MS/MS analysis. Secondary ions were isolated within a 2.0-*m/z* window.

### MS/MS data interpretation and label-free protein quantification

The MS/MS spectra recorded for each sample were assigned to peptide sequences using the MASCOT Server 2.5.1 (Matrix Science). Contaminant spectra were first removed by searching against an in-house “common contaminants” database (384 sequences; 187,250 residues) including 361 contaminants classically observed in proteomics (cRAP + additional contaminants) and 23 *Bos taurus* sequences corresponding to the most abundant proteins from FCS, present in the cell culture medium [18]. The remaining sequences were searched against a database (20,585 sequences; 10,847,418 residues) containing the UniProt *Chlorocebus* sequences (downloaded March 2020) and the Italy-INMI1 SARS-CoV-2 protein sequences. The following parameters were used in peptide-to-MS/MS spectrum assignment: full trypsin specificity, maximum of two missed cleavages, mass tolerances on the parent ion of 5 ppm and 0.02 Da on the MS/MS, static modifications were carbamidomethylated cysteine (+57.0215) and oxidized methionine (+15.9949), whereas the dynamic modifications allowed were deamidation of asparagine and glutamine (+0.984016). MASCOT DAT files were parsed using the Python version of Matrix Science msparser, version 2.5.1, with the ms_peptidesummary function. Peptide-to-Spectrum Matches (PSMs) with expectation values corresponding to a 1% False Discovery Rate (FDR) were validated using MASCOT’s homology threshold option. Multiple PSMs were allowed for MS/MS spectra if ion scores were higher than 98% of the top ion score. Proteins were grouped if they shared at least one peptide, and label-free quantification of each group was based on PSM counts for each protein following the principle of parsimony. Proteins identified by one or more specific peptides were retained for the analysis (protein FDR 1%).

### MS/MS data repository.

Mass spectrometry proteomics data have been deposited to the ProteomeXchange Consortium via the PRIDE [19] partner repository under dataset identifier PXD018594 and 10.6019/PXD018594.

### Data analysis

Principal component analysis was performed as previously described [20]. Co-expression cluster analysis was performed using the Bioconductor R package coseq v1.5.2 [21]. The protein abundance matrix was used as input in coseqR. Log CLR-transformation was applied to the matrix to normalize the protein abundance profiles and the K-means algorithm was chosen to detect clusters that were co-expressed across the different time-points. The K-mean algorithm was repeated 20 times to determine the optimal number of clusters. The resulting number of clusters in each run was recorded, and the most parsimonious cluster partition was selected using the slope heuristics approach. Both the principal component analysis (PCA) and the coseq analysis were performed after removal of proteins with spectral counts of less than three (1402 host protein groups retained). Finally, proteins assigned to the different clusters were retained for cluster visualization and gene ontology (GO)-enrichment analysis for each cluster. Statistically enriched (FDR ≤ 0.05) GO terms on proteins that are differentially expressed between pairwise samples or on proteins assigned to each co-expression cluster were identified using Metascape [22]. The statistically most enriched GO terms were visualized in ggplot2 [23].

## Results

### Profiling virus production by tandem mass spectrometry

**Figure 1.**
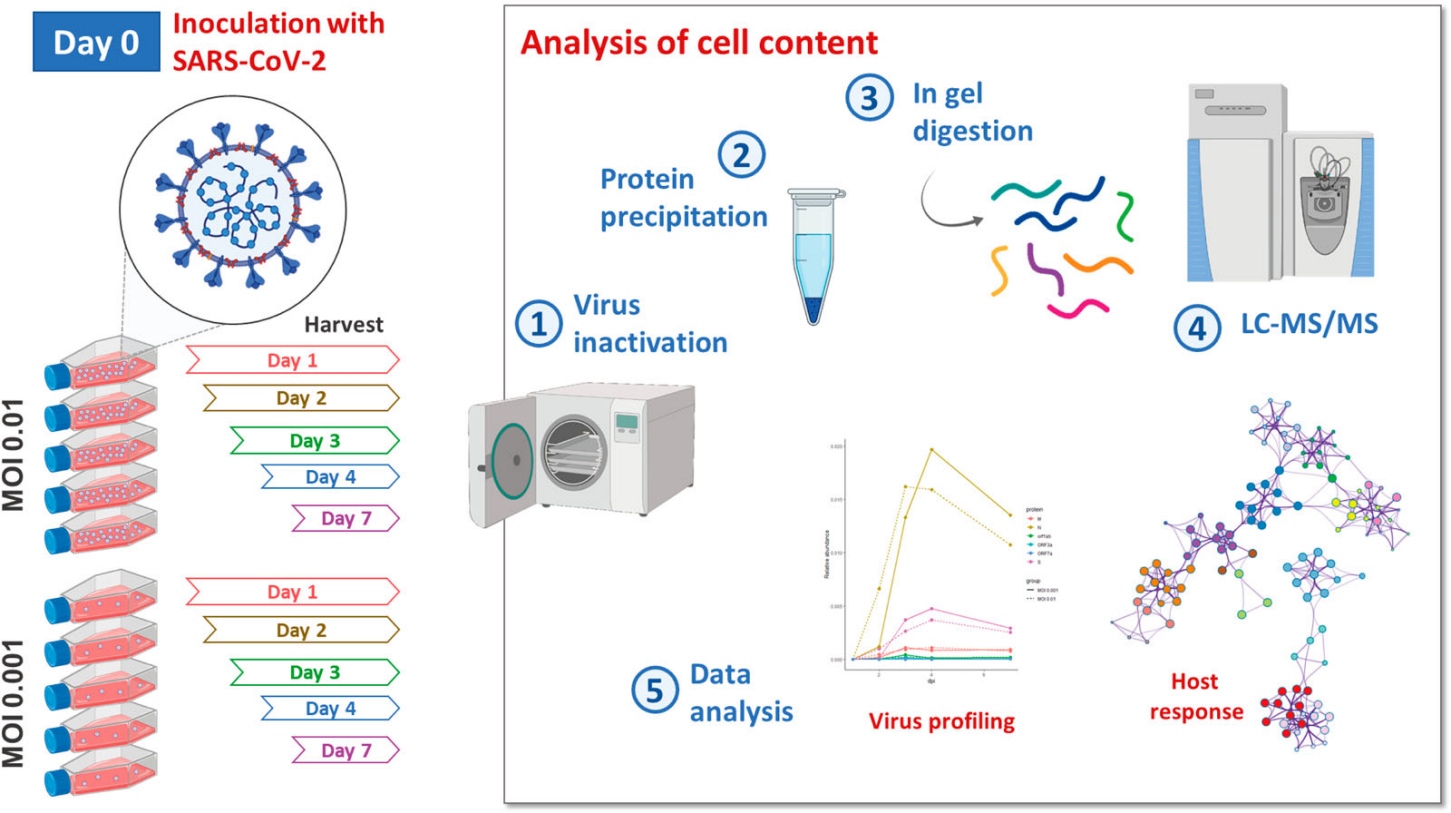
Schematic representation of the experimental design. Vero E6 cells were infected on Day 0 with SARS-CoV-2 at two multiplicities of infection (MOI; 0.01 and 0.001). The kinetics of the infection was monitored by tandem mass spectrometry over several days. The main steps and the output of the analysis are highlighted.

To understand the dynamics of SARS-CoV-2 infection and determine optimal conditions for whole viral particle antigen production, we infected Vero E6 cells with SARS-CoV-2 at two MOI (0.01 and 0.001) and monitored the kinetics of infection over several days using tandem mass spectrometry (Figure 1). As the significant biological characteristics of infectious SARS-CoV-2 in tissues are currently unclear, a delayed time-point of 7 days post-infection was included to understand the relationship between virus replication and cell damage, which appeared quite mild at early time-points. Overall, 3220 Vero cell proteins and 6 SARS-CoV-2 proteins were identified by 27,388 and 94 unique peptides (FDR < 1%), respectively (Table S1). Among the viral proteins, three (N, S, M) of the four structural proteins, and three of the sixteen non-structural proteins were identified. The non-structural proteins were peptides corresponding to the ORF1a papain-like protease (PLpro) / 3C-like protease (3CLpro) and the accessory proteins encoded by ORF3a and ORF7a. These protein sequences were covered by 40 (N), 29 (S), 7 (M), 13 (ORF1ab), 4 (ORF3a), and 1 (ORF7a) distinct peptides. Sequence coverage for polypeptides depends on their abundance, size, and the occurrence of lysine and arginine residues. The ORF7a protein, with just 121 residues, was thus logically not as well detected as the others. Based on the sets of peptides shared by the individual proteins, 2984 protein groups were identified, for which a total of 457,111 PSMs were assigned. Viral proteins represented 1.4% of these PSMs.

**Figure 2.**
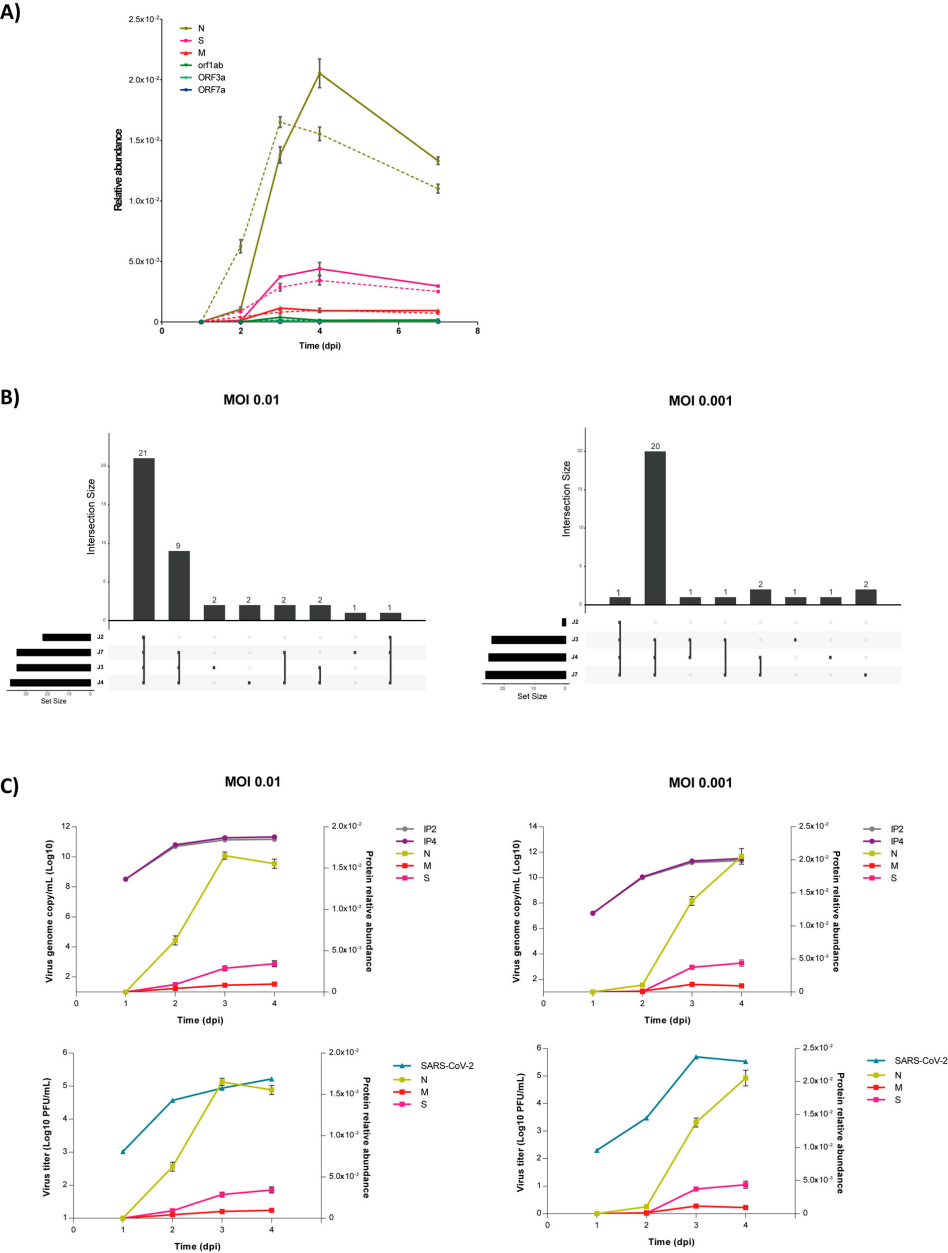
Virus profiling by tandem mass spectrometry. **(A)** Kinetics of viral production revealed by LC-MS/MS. Relative abundance at each time-point represents the mean ± standard deviation of the technical replicates at the two MOI tested. Dashed curves indicate results obtained at MOI 0.01; solid curves refer to MOI 0.001. **(B)** Comparison of viral peptides identified at the different time-points. Sets of intersections were visualized using the UpSet matrix layout and plotted horizontally. Each column corresponds to an exclusive intersection containing the elements of the sets represented by the dark circles. Sets are represented by the peptides assigned to viral proteins at each time-point. J2, J3, J4 and J7 refer to the different time-points analysed: Days 2, 3, 4 and 7, respectively. **(C)** Correlation between the abundance of viral proteins measured by LC-MS/MS across time-points and number of SARS-CoV-2 genome copies (top panel) and virus titre (bottom panel) obtained at MOI 0.01 and 0.001.

The dynamics of viral protein levels across time-points indicated that SARS-CoV-2 protein synthesis increased continuously after infection, with a peak registered on around day 3 post-infection. Viral amplification was slightly delayed in cells infected at MOI 0.001 (Figure 2A). SARS-CoV-2 N, S, and M were consistently among the most abundant proteins detected. Their respective ratios were relatively constant over time. The decline in abundance of viral proteins registered at 7 days post-infection was in accordance with notable cytopathic effect following intense virus replication, observed here for all cultures at this time-point, as previously reported [24].

As expected, a larger variety of peptides was detected for overrepresented proteins. Figure 2B shows the distribution of this diversity across the time-points for the three most abundant viral proteins. Interestingly, increased protein levels was reflected by increased abundances of the same set of peptides, with only a few new sequences recorded at the peak of viral production compared with an early time-point.

To determine to what extent virus profiles obtained by tandem mass spectrometry reflected viral particle production, we measured SARS-CoV-2 RNA molecules by quantitative PCR and virus titre at the same time-points (Figure 2C). Variations in most abundant viral protein yields were consistent with variations in the number of SARS-CoV-2 RNA molecules and the amount of infectious virus particles, confirming that LC-MS/MS with label-free quantitation can be applied to monitor SARS-CoV-2 infection kinetics.

### Characterization of host cell protein dynamics upon SARS-CoV-2 infection

**Figure 3.**
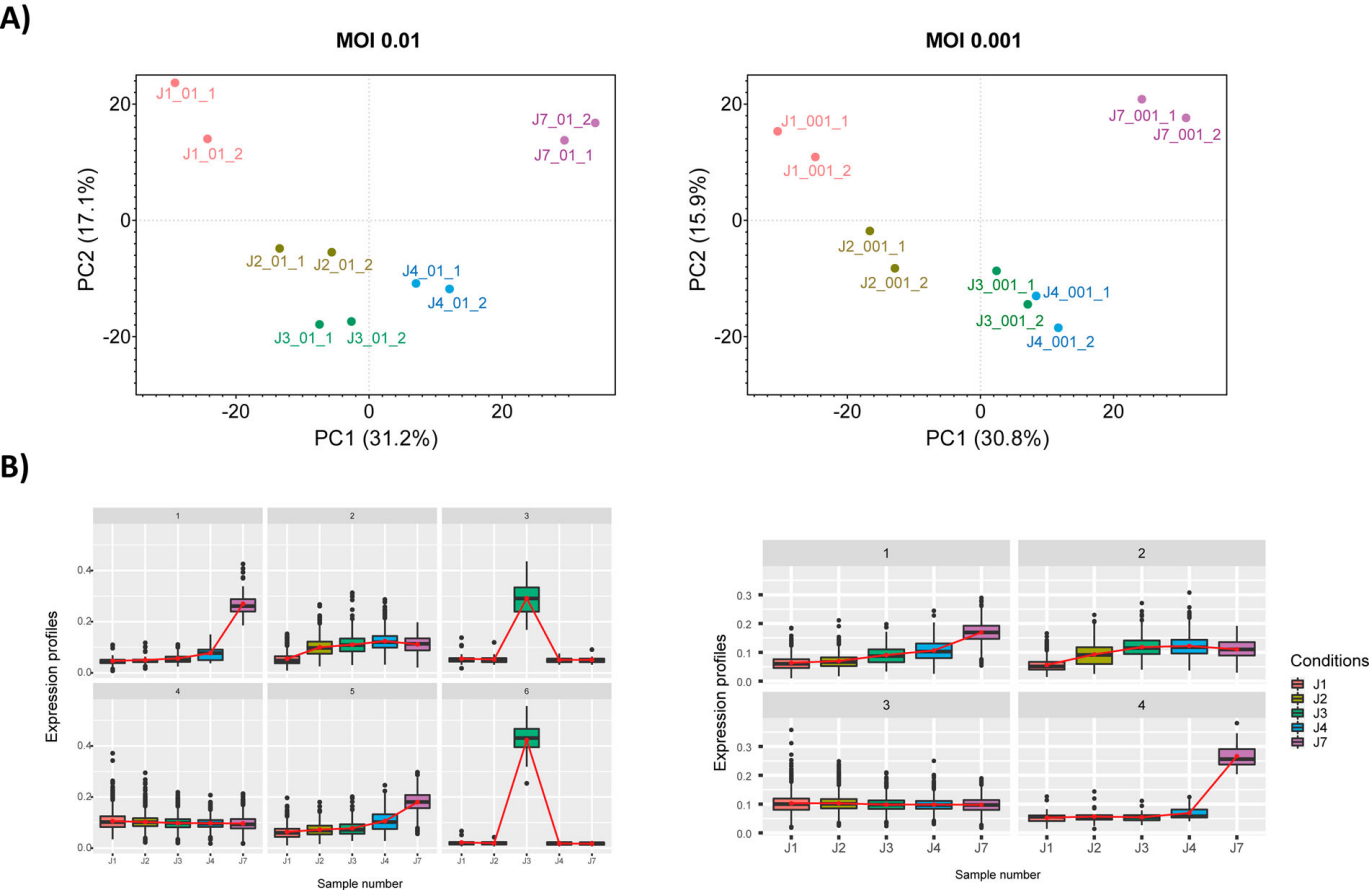
Host response following SARS-CoV-2 infection. **(A)** Dimension reduction by principal component analysis (PCA) of the different time-points for MOI 0.01 and 0.001. The normalized abundances of proteins with Spectral Count >3 was used as input. J1, J2, J3, J4 and J7 refer to the different time-points analysed, Days 2, 3, 4 and 7, respectively. **(B)** Clusters of proteins showing similar expression profiles over time. J1, J2, J3, J4 and J7 refer to the different time-points analysed, Days 2, 3, 4 and 7, respectively. The number on the top of each plot identifies the cluster.

We next characterized changes in cellular protein networks upon SARS-CoV-2 infection at the level of total protein abundance. Dimension reduction by PCA showed that early and late time-points were distinctively distributed along principal components 1 and 2, with replicates clustering closely. The same pattern was observed for both MOI. Interestingly, a degree of overlap was observed for time-points J3 and J4, corresponding to the peak of viral protein abundance, suggesting a smaller evolution of the host proteome between these time-points (Figure 3A). Addition of the viral proteins measured at each time-point did not affect sample separation. To elucidate the host response during virus amplification, we performed a co-expression cluster analysis to determine host proteins showing similar profiles over time (Figure 3B). Functional enrichment analysis performed on the members of each cluster provided an overview of the biological processes affected by the virus (Figure 4A-C). At MOI 0.01, clusters 2 and 5, with 345 and 198 protein members respectively, had very similar statistically enriched terms, with functions and pathways related to the viral life cycle among the most frequently represented. Functional interaction network analysis showed cluster-specific enrichment of functions like membrane trafficking and protein pre-processing in the endoplasmic reticulum, to be highly interconnected with regulation of mRNA processing/splicing via spliceosome (Figure 4A-B). In addition, enriched biological functions overlapped for clusters 3 and 6, with 16 and 10 representatives, respectively, characterized by a specific increase in the abundance of clustered proteins at 3 days post-infection. Proteins involved in cornification and ECM (Extra Cellular Matrix) regulators were among the top 20 significantly enriched terms (Figure 4A). Four distinct expression profiles were identified at MOI 0.001 (Figure 3B). Comparative analysis and inference of enriched biological pathways revealed significant enrichment of functions related to host responses to viral replication in clusters 2 and 3. Thus, clathrin-mediated endocytosis (R-HSA-8856828) and vesicle-mediated transport (R-HSA-5653656) pathways were specific to cluster 2 (Figure 4D); these pathways are likely to be involved in vacuole formation and viral budding. Interestingly, similar to cluster 2 identified at higher MOI, the expression profiles of the members of these clusters reflect the dynamics of viral protein levels across the kinetics. Clusters 4 (MOI 0.01) and 3 (MOI 0.001) were enriched in pathways and biological functions related to the host cell’s central metabolism, with RNA metabolism and translation among the most highly enriched pathways. Although the temporal expression profiles of the proteins in these clusters show no major changes, we cannot exclude that they are remodelled by the virus, or that their abundance is drastically altered at earlier time-points (<24 h).

Figure 4.Host pathways deregulated by the infection. **(A,C**) Heatmaps showing the main enrichment clusters, one row per cluster, using a discrete colour scale to represent statistical significance. Grey indicates a lack of significance. MOI 0.01**(A)** and 0.001 **(C). (B,D)** Enrichment network visualization for results from the proteins present in each of the clusters identified. Nodes are represented by pie charts indicating their associations with each cluster. A colour code represents the clusters. At MOI 0.01 **(B),** red represents Cluster 1; blue, green, purple and orange refer to Clusters 2, 3, 5 and 6, respectively. At MOI 0.001 **(D),** red indicates Cluster 1; blue and green refer to Clusters 2 and 4, respectively.

**Figure:**
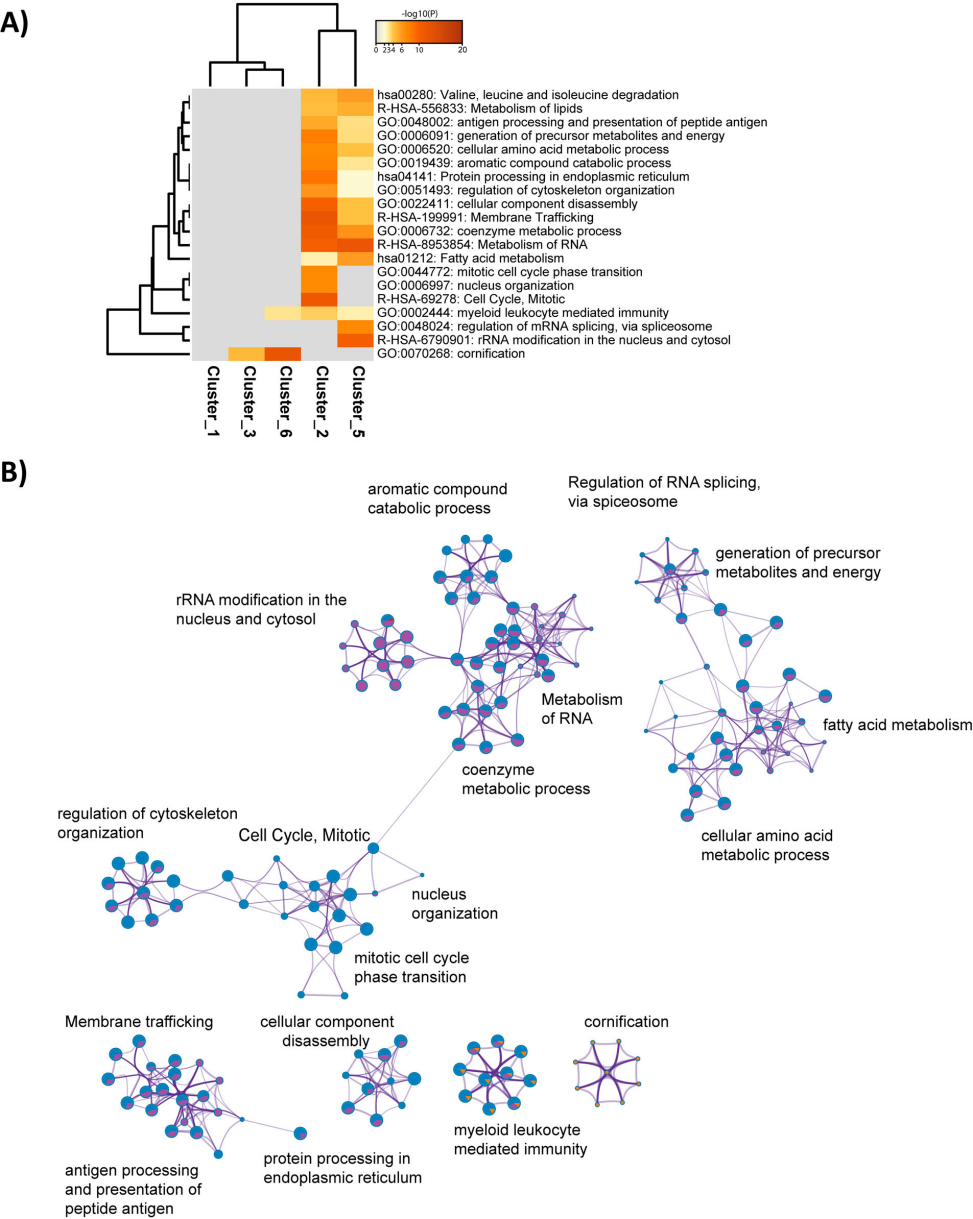

**Figure:**
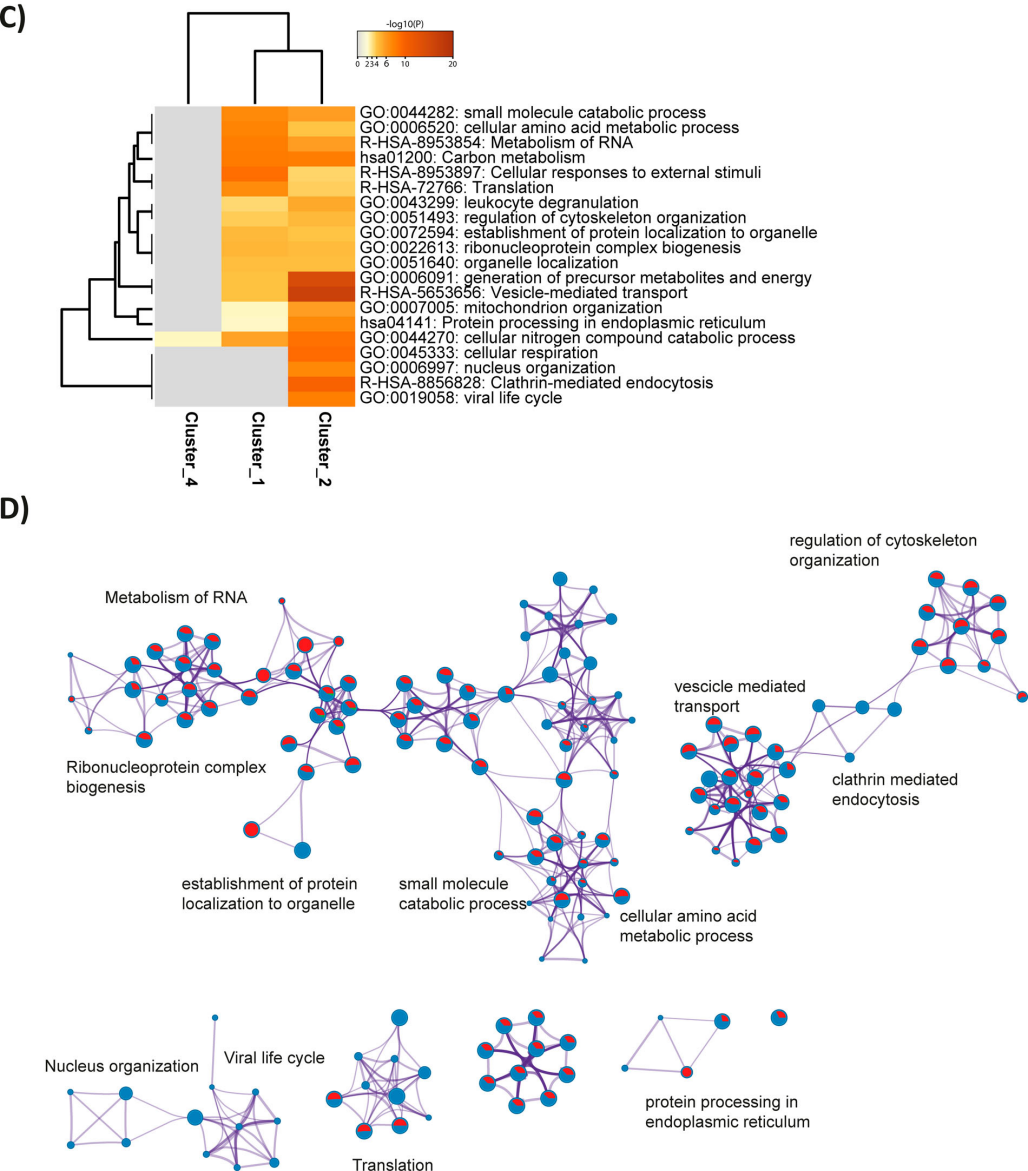

## Discussion

In the race to develop a vaccine to control the spread of SARS-CoV-2 worldwide, a number of technology platforms have been tested [5]. In this context, in the search for vaccines based on inactivated or attenuated whole viral particles, the identification of the right conditions for virus amplification could be challenging. Virus yields from dedicated cell culture systems could also represent a limitation. Alongside vaccine development, inactivated virus particles are also of interest when testing real serology or screening neutralizing antibodies. Naturally, production of well-characterized active virus particles is also of interest for basic research purposes. Given the requirement for speed, for this study we evaluated the use of LC-MS/MS as a tool to guide condition optimization for SARS-CoV-2 whole viral particle antigen production.

The results presented here demonstrate the potential of our pipeline to profile virus production over time. In particular, by analysing the proteome of Vero cells infected with SARS-CoV-2 at two different MOI, it was possible to monitor changes in the levels of three SARS-CoV-2 structural proteins and three non-structural ones. As for other analyses [13,15], peptides from protein E-like were undetectable. The lack of evidence for other accessory proteins could be put down to differences in sample processing, with the protocol described here favouring simplified steps and speed, while maintaining accuracy. More extensive analyses are planned to monitor virus homogeneity during viral production once the most permissive conditions have been established.

Protein profiles were remarkably comparable at the two MOI tested, with the profiles obtained at the lower MOI slightly delayed and hence more insightful regarding the timing of the viral burst.

Overrepresented proteins were identified by a larger variety of peptides. Interestingly, an increase in protein levels was reflected by the increasing abundance of the same set of peptides, with only a few new sequences registered at late time-points. This observation suggests that targeted absolute quantification of the virus could be used to monitor peptides that are detectable early. Tandem mass spectrometry proteotyping [25,26] could also ultimately be proposed to detect SARS-CoV-2 viral activity.

In addition to profiling virus production, our mass spectrometry analysis of the whole cell content provides insights into the cellular response to SARS-CoV-2 infection. Although more detailed information is available regarding how the virus enters the cell, increased information on the subsequent steps in the SARS-CoV-2 replication cycle are needed [27]. We are not aware of other attempts to characterize the cellular response to infection with SARS-CoV-2 in Vero E6 cells. These cells mount a weak innate response to viruses, making them extraordinarily competent for propagating several virus types to high titres [28]. However, this specificity means that similar proteomics data should be acquired for multiple cell lines from humans and primates, using comparative proteomics to define the shared mechanisms of cell infection and those that are specific to a given cell line. This approach will allow us, ultimately, to grasp all the essential aspects of the host cell response to SARS-CoV-2 infection.

The analysis of our proteomics data suggested substantial temporal remodelling of the host proteome over the time-points analysed. Functional enrichment analysis of clusters of proteins showing similar expression profiles highlighted key pathways during virus replication. The result provides potential targets for effective therapies against coronaviruses. In corroboration of the pathways identified by Bojkova et al. [13] using the human colon epithelial carcinoma cell line Caco-2 to analyse SARS-CoV-2 replication, we identified clusters of proteins increased by infection that were particularly enriched in RNA modifiers, such as spliceosome components, and proteins involved in carbon metabolism, further supporting the preliminary evidence that splicing is an essential pathway for SARS-CoV-2 replication, and thus a potential therapeutic target. Additional clusters of proteins identified in our analysis highlighted the regulation of pathways critical for the virus life cycle, such as those involved in protein pre-processing in the endoplasmic reticulum, vacuole formation and viral budding. Interestingly, proteins participating in viral RNA replication and translation or involved in various functions including cytoskeleton dynamics, metabolic enzymes, and signal transduction were described in 2005 by Jiang and colleagues [29] as differentially regulated after SARS-CoV infection of Vero cells.

Here, we show that our pipeline based on LC-MS/MS analysis is suitable for the characterization of SARS-CoV-2 production. We therefore suggest that when optimizing the conditions for viral amplification, it could be relevant to speed up the initial steps in favour of later stages during the development process, which will require a more careful assessment of effectiveness and safety.

In addition, the peptide information presented here provides sufficient information to design targeted analyses [30], opening the possibility of using targeted mass spectrometry-based approaches to assess critical aspects (*i.e.* quality and quantity) during virus purification processes. Furthermore, the changes characterized in cellular protein networks following SARS-CoV-2 infection provided valuable avenues for further exploration and could guide the identification of drug targets to address the pandemic caused by SARS-CoV-2.

We anticipate that the same workflow could be successfully applied to expedite the characterization of human organ-on-a-chip (Organ Chip) microfluidic culture devices used to obtain insights into the successive steps in the virus’ life cycle, as well as to study human disease pathogenesis [31] in response to infection by variants of SARS-Cov-2 and following the application of existing [32–34] and novel therapies.

## Author contributions

LG, FG, OP, LB, and JA conceptualized the study design; FG, JCG, HB, SR, and NB performed the experiments; LG, OP, DG, FG, LB and JA analysed the data; GM, KC, SD, MAR, GS, CF, AD, BAB, and CA contributed reagents and to software development; LG and JA drafted the manuscript. All authors read and approved the final manuscript.
